# Supplementary material for: Complexome profiling of the Chlamydomonas psb28 mutant reveals TEF5 as an early PSII assembly factor
Source: Plant Cell. 2025 Mar 17;37(6):koaf055. doi: 10.1093/plcell/koaf055 (PMC12164590; doi:10.1093/plcell/koaf055)
Supplement: koaf055_Supplementary_Data [file koaf055_supplementary_data.zip › psb28_interactive.zip!/plots/intensity/qualityPlots/peptides/OHP1_Cre02.g109950.t1.2.html]

### Cre02.g109950.t1.2

Trivial name: OHP1  
SeqCoverage [median]: 29.60  
Mass: 11.39 kDa  
  
 

| Sequence | MissedCleavage | Mass [Da] | wtIntensity | muIntensity | Score |
| --- | --- | --- | --- | --- | --- |
| AAVVPAPK | 0 | 751.46 | 0.00 | 314.22 | 79.45 |
| GISMPPK | 0 | 728.39 | 0.00 | 775.43 | 94.16 |
| GISMPPKQPDVPPPK | 1 | 1586.85 | 9277.56 | 12845.02 | 116.96 |
| NGFVDYAER | 0 | 1069.48 | 0.00 | 4094.96 | 112.41 |
|  |  |  | 9277.6 | 18029.6 |  |
